# Becoming a Distance Manager: Managerial Experiences, Perceived Organizational Support, and Job Satisfaction During the COVID-19 Pandemic

**DOI:** 10.3389/fpsyg.2022.916234

**Published:** 2022-08-18

**Authors:** Christine Ipsen, Kathrin Kirchner, Nelda Andersone, Maria Karanika-Murray

**Affiliations:** ^1^Management Science Division, Department of Management, Technical University of Denmark, Kongens Lyngby, Denmark; ^2^Department of Psychology, School of Social Sciences, Nottingham Trent University, Nottingham, United Kingdom

**Keywords:** work extensification, leaders, well-being, working from home (WFH), pandemic (COVID-19), distance management, Hybrid-work, HRM

## Abstract

Due to the COVID-19 pandemic having radically changed the way we now work, many recent studies have focused on employees’ experiences and well-being, their performance and job satisfaction (JSA), and ways to ensure the best support for them when working from home (WFH). However, less attention has been given to managers’ experiences in adapting to the new role of distance management and supporting them with this transition. This study aims to explore how managers experienced distance management, and the perceived organizational support (POS), and the effect of organizational support has on their JSA during the first year of the COVID-19 pandemic. Data from 1,016 line, middle and top managers in Danish workplaces were collected in March 2021, 1 year after the start of the COVID-19 pandemic. We applied descriptive statistics, ANOVA, and partial least square structural equation modeling to investigate the relation of perceived organizational support (POS) and the JSA of distance managers. Control variables were the respondents’ demographic characteristics, specifically gender, age, and management level. The study offers insights into the managerial experiences of becoming a distance manager, helps to understand the relationship between POS and managers’ JSA, and shows a positive relationship similarly for the managers as for employees. The study shows that most managers found their work as distance managers more demanding and worked more hours. The data demonstrate that managers received the most support from their own employees and manager peers, whereas administrative support was largely lacking. The data also show that the majority (67%) of the managers prefer to manage from the office, but similarly, they can continue managing from a distance if needed post-pandemic. The study adds to the literature on workplaces’ transitioning to distance management and hybrid work and contributes to understanding the role of POS and managers’ JSA during this transition. Consequently, if an organization aims to offer hybrid work, improving support from top management and in-house support functions would help maintain or increase managers’ JSA.

## Introduction

When the COVID-19 pandemic struck the world in 2020, it led to severe job changes for both employees and managers for whom distance work and distance management became compulsory, as governments across the world required people to work from home to avoid the spreading of the virus (BBC, 2020; WHO, 2020a). During the last few decades, digitization has permitted practices such as remote work and telework (i.e., working from home, WFH) (Kurland and Bailey, 1999; Markus et al., 2000; Gurstein, 2001; Bailey and Kurland, 2002). However, prior to the pandemic these practices, were still relatively new to most organizations (Vargas, 2020). Consequently, this led to a strong focus on ensuring employees’ well-being when WFH, and a considerable amount of research was conducted focusing on employees’ experiences and behaviors in the change to work from home during the pandemic. Several studies have shown that WFH during the pandemic came with both advantages and disadvantages for employees (Carnevale and Hatak, 2020; Graves and Karabayeva, 2020; Ipsen et al., 2021) and can drive increases in both performance and productivity (Apollo, 2022; Weber et al., 2022).

Studies indicate that an increasing portion of the workforce will continue to work from home after the pandemic (Gartner, 2020b; Lister, 2020) Thus, distance management will continue to be an important issue in the discussions surrounding “the future of work.” Although the pandemic has affected both employees and managers, most of the discussions have focused on the role of organizational support towards employees and ways they have been influenced by different factors (see for example (Weber et al., 2022). As organizations continue to discuss how to continue WFH post-pandemic (Gartner, 2020a; The Economist, 2020; The European Commission’s Science and Knowledge Center, 2020; Predotova and Vargas, 2021), it is essential to understand how managers experienced the change in their role and the transition phase and eventually what would be required of them to act as distance managers. Hence, additional studies are needed to gain insight into their experiences and the consequences of managers’ work extensification to understand the POS among managers and if it influenced their motivation and job satisfaction (JSA) related to becoming distance managers. This would allow creating a pathway for utilizing organizational support in practice, for those acting in a management role and as a distance manager. Therefore, the questions in focus are: During this transition, how did managers perceive becoming distance managers and in particular what support did they get and from which actors? Additionally, did perceived organizational support (POS) have an effect on their job satisfaction? And finally, does the managerial level/seniority have an effect on these experiences?

Consequently, the aim of the current study is to contribute to the literature by understanding i) how managers across organizational levels experienced their new role, i.e., becoming a distance manager and ii) examining their POS across three management levels and iii) to understand whether POS is related to their motivation and JSA. Building on the analysis of the data collected among members of the Danish Association of Managers and Executives, the paper also discusses challenges related to the future of work, which is now taking a more hybrid form worldwide focusing on the implications for human resource management (HRM) and management in general. In addition, we propose avenues for research and advocate for a joint research agenda to mitigate the challenges identified.

## Literature Review

“Telecommuting,” “telework,” and “virtual organizations” (Van der Widen et al., 1993; Handy, 1995; Lamond et al., 1997) imply remote working, primarily through the use of different technologies, as a substitute for physical traveling (Nilles et al., 1976). Studies have found that the flexibility offered by distance work, telework, and WFH, include an entire range of advantages and disadvantages for employees, employers and organizations (Kurland and Bailey, 1999; Bailey and Kurland, 2002; Charalampous et al., 2019).

The increasing interest in the effect of WFH during the pandemic has heightened the need to understand how managers should manage and lead hybrid work from a distance to support their employees’ mental health and performance (Giurge and Bohns, 2020; WHO, 2020b; Gratton, 2021). The current literature has paid little attention on managers’ experience with distance management broadly, or specifically their POS and how that support may relate to their JSA and motivation.

In the long-term, manager’s reduced JSA and motivation can impact employee well-being, as studies show that managerial stress affects employees negatively (Skakon et al., 2010, 2011). Therefore, there is a need to focus on how organizations can support their managers in the transition to distance management. If workplaces increase the use of WFH post COVID-19, distance managers must acquire new skills and accept the change in the nature of their job. Such needed knowledge will allow us to understand the challenges that distance managers face and enable the development of guidelines on supporting distance managers.

### Managerial Work and Work Extensification as Distance Managers

Before the pandemic in 2019, there were large differences in the prevalence of telework across the European Union’s (EU) Member States. Only 5.4% of employed in the EU-27 reported that they worked from home “usually.” The prevalence of telework varied strongly across sectors and occupations. It was exceptionally high in knowledge- and Information and Communication Technology (ICT)-intensive services. In Denmark, for example, 20% of the total workforce would “sometimes” work from home, whereas 5–10% would “usually” work from home (The European Commission’s Science and Knowledge Center, 2020). Undeniably, digital technology empowers people with greater flexibility, yet it also lures and confines with its connectivity. Research links the use of digital technology with enhanced worker engagement, work overload, availability expectations, and blurring of professional and private lives, thus affecting their well-being, motivation, and JSA (Hassard and Morris, 2021).

While providing more flexibility and autonomy to employees, the pandemic’s restrictions and people WFH also led managers to work more, having greater responsibilities and experiencing the need for more devotion to work (Hassard and Morris, 2021). COVID-19 and consequential distance work has further extensified work for employees, including longer work days with more tasks, and responsibilities (Kost, 2020). In the case of managers, an early COVID-19 study of their mental health showed that many also experienced higher anxiety and depression (Graf-Vlachy et al., 2020), and a lack of trust (Parker et al., 2020). A Danish study found that managers experienced WFH as more challenging than the employees (Ipsen et al., 2021).

According to Worrall and Cooper (2007), the number of hours managers deliver over and above their contract hours has increased in parallel to the initiatives designed to improve employees’ work-life balance. Similar considerations have been taken to employees during the pandemic, and the question is whether this has impacted the manager’s working hours, i.e., extensified their work. Therefore, we are concerned with the extensification of managerial work as a basis for understanding the implication for the new ways of working and managing.

Work extensification entails the distribution or exporting of work across different spaces/scales and times to deal with high workloads and enable the completion of work tasks. In their study of managerial work in the digital age, Hassard and Morris (2021) defined situations “where changes to work reflect two trajectories: (i) where the contractual employment boundary becomes stretched as hours completed inside the workplace increase; and/or (ii) where this boundary becomes breached by managers voluntarily conducting work from locations outside corporate premises” (p. 2). They state that extensification’ captures the dynamic of the modern managerial labor process more holistically than work intensification” (p. 2).

Consequently, to understand extensification in managerial work during the pandemic, there is a need to gain insight into managers’ experiences with distance management and how they adapted to the role as distance managers. Through the COVID-19 pandemic, there has been an ongoing discussion about the benefits and positive effects of WFH. One of these benefits concerns the increased efficiency and performance related to WFH. However, it is increasingly important to investigate whether managers have also experienced similar benefits or whether the changes merely led to extensification of their work.

### Managers’ Perceived Organizational Support and Job Satisfaction

Organizational support during a change in managerial work practices is an essential factor in JSA. Organizational support theory has received a lot of attention in examining the employee–employer relationship (Eisenberger et al., 1986, 2002), and research has consistently showed the advantages for organizations of having people that feel supported. According to Eisenberger et al. (1986) individuals’ generalized perception of their organization’s ability to care for and support them can affect their attitudes and behaviors. Consequently, according to the POS theory, peoples’ perception of their organization’s ability to support, care and react to their needs forms the basis of their opinions. Thus, POS has been found to relate to higher JSA and increased performance (Caesens et al., 2016), commitment and reduced turnover (Maertz et al., 2007), and reduced stress. Furthermore, those with higher levels of experienced POS are more committed to the organizations they work for and more satisfied with their jobs (Rhoades and Eisenberger, 2002).

In circumstances when people are engaged in new ways of working, such as teleworking or WFH, support from an organization has especially a large impact on feelings of appreciation, engagement, and work behaviors (Eisenberger et al., 1986, 1997, 2002; Biron et al., 2021). Although it is important to acknowledge that first-line and middle managers as employees with distinct responsibilities including decision-making and leading others (Kraut et al., 1989), existing POS studies have focused primarily on employees without such responsibilities. Management and leadership during changes and interventions is a dynamic role where leaders must develop and be supported in tandem with an organizational change or intervention (Ipsen et al., 2018). In the transition to becoming a distance manager during and post the COVID-19 pandemic, studies have unacknowledged the managers’ experiences and their perception of organizational support. The question is how managers have been supported in tandem with the change in their role, and the increased flexibility enabled by telework, allowing people to work from home. Furthermore, despite the early observations of managers being challenged by their new roles and the changes in their job when people are WFH, the mechanism has remained unclear.

In the light of the above, we aimed to examine the consequences of managers’ work extensification on managers’ experiences of POS on managers’ motivation and JSA. Just as POS has been found to be related to numerous effects on employees and yield positive consequences for organizations, we argue that the same relationship should occur at the management levels. The interest in JSA related to the new ways of working has highlighted the importance of understanding the role of POS, which can help identify the actions that workplaces and HRM need to take to ensure JSA and motivation among all managers. Thus, we focus on examining the effects of managers’ POS on managers’ motivation and JSA. Based on the above, we hypothesized that:

**Hypothesis 1:** POS is positively related to managers’ JSA when becoming a distance manager.

The literature on managers involves a wide variety of job settings and types. Given the broad scope of the existing research, we focused on aspects most relevant to this study: how managerial tasks differ across management levels. Literature and practice typically differentiate between three management levels: strategic (top managers), tactical (middle managers), and operational levels (line managers), whereby, depending on the management level, managers perform different tasks. Strategic/top managers are responsible for developing the organization’s strategy and defining the vision and mission. They ensure the organization’s competitiveness, whereas middle managers are responsible for the efficiency and effectiveness of an area. Line managers lead a function that contributes directly to the products or services the organization creates.

Building on the work by Fayol (1949) and Carroll and Gillen (1987), the principles of management have long been categorized into four major functions. These functions are planning, organizing, leading, and controlling (the P-O-L-C framework) to achieve business goals effectively and efficiently (Jones et al., 2019). Although these functions have been criticized for being too fixed (Lamond, 2004; Pistrui and Dimov, 2018), this approach to management enables it to be applied at different management levels where the mutual weighting between the different management functions differ (Jones et al., 2019). According to Jones et al. (2019), the vertical divide of an organization in a hierarchical format and the management functions divided horizontally by management levels implies that managers at the same level have similar duties, tasks, and authority (Jones et al., 2019). However, although managers have responsibility in all the four functions, they do not exercise these in equal amounts (Mintzberg, 1971). Where first line managers spend most of their efforts on leadership compared to top managers, the latter spend most time on planning and organizing. Middle managers balance all four functions, and spend less time than top managers on planning and organizing but more time on leading and controlling than first line managers.

As managerial tasks vary across management levels in general; this can also be assumed to happen during transformations like changing work into hybrid work where managers became distance managers. Following Hypothesis 1, the question is whether the experiences and perception of the organizational support vary by managers’ seniority (Worrall and Cooper, 2007).

## Materials and Methods

### Design and Sample

To assess managers’ experiences of work extensification in the context of distance management when employees were WFH, we applied questions about the managers’ time spent working when WFH and working beyond their agreed time (Worrall and Cooper, 2007; Currie and Eveline, 2011; Faragher et al., 2013) and adjusted the items to include the context of distance management. We used a 5-point Likert scale (1 strongly disagree, 2 disagree, 3 undecided, 4 agree, 5 strongly agree) to assess how respondents perceived their work. This covered three (3) items: “I work more hours per day when I work from home”; “I take less breaks when I work from home”; “I have problems to separate work and family life when I work from home” similar to items in the ASSET questionnaire (Worrall and Cooper, 2007).

To gain a balanced view of the experiences of distance management, whether they also experienced benefits when WFH, we included an item on performance: “I am more productive when I work from home” (Bailey and Kurland, 2002; Ipsen et al., 2021; Weber et al., 2022). To examine elements within managers’ POS that may lead to JSA or lack of satisfaction, this study used a partial least square structural equation model (PLS-SEM). The hypothesis in our research model was validated by a survey administered among Danish managers. The survey included demographics and 30 questions connected to distance management. Using an online survey, we collected data from members of the Danish Association of Managers and Executives (DAME), Lederne, in March 2021 when restrictions were lifted, and the numbers of COVID-19 cases were decreasing.

A total of 1,016 managers provided fully completed questionnaires. Multiple item scales were employed to measure the latent variables. The items for POS and JSA were measured on a 5-point Likert scale ranging from 1 (totally disagree) to 5 (totally agree). Measurement items for the latent constructs POS and JSA were adapted from the work of Caesens and Stinglhamber (2014), the Brief Overall Job Satisfaction Measure II (Eisenberger et al., 1986; Judge et al., 1998). We first accounted for managers’ attitudes toward the organization and their new role as distance manager with a five-item measure of JSA. These five items comprised JSA, individuals’ positive feelings or attitude toward their overall job; motivation, wellbeing, and personal development, where the POS items included support (horizontal and vertical), acknowledgment and the ability to develop in the context of distance management. Examples of items are “I experience that I get support from my workplace (e.g., HRM/Internal support) in my role as distance manager” or “I find that my workplace appreciates/recognizes my efforts as a distance manager.” Furthermore, we collected demographic information.

Table 1 gives an overview of the demographics of the whole sample and of the three investigated management levels. Only managers working fully or partly from home during data collection were included. Most managers were working in the private sector (76.8%). A total of 69.5% of managers did hybrid work and shifted between the office and home office, while 59.8% of their employees worked partly home and partly from the office. For line managers, the gender of survey respondents was balanced, while more men were answering on the higher levels of management. The higher the management level, the older was the survey participants. The majority of top managers came from private and self-governing organizations and from smaller companies.

**TABLE 1:** Characteristics of the investigated managers.

Demographics	All (*N* = 1,016)	Line managers (*N* = 480)	Middle managers (*N* = 356)	Top managers (*N* = 180)
**Gender**				
Female	40.6%	50.2%	33.1%	30%
Male	59.4%	49.8%	66.9%	70%
**Age**				
18–29	0.8%	1.3%	0.6%	0%
30–39	9.4%	12.5%	6.7%	6.1%
40–49	33.1%	34.4%	33.7%	28.3%
50–59	47.1%	43.1%	50%	52.2%
60 or more	9.6%	8.8%	9%	13.3%
**Working from home**				
Only work from home	30.5%	36.5%	28.4%	18.9%
Partly work from home	69.5%	63.5%	71.6%	81.1%
**My employees work**				
Constantly/always and fulltime from home	0.5%	0.8%	0.3%	0%
Only from home right now	23.7%	30%	19.9%	14.4%
Only from their workplace	13.4%	14.2%	15.7%	6.7%
Are sent home and do not work	2.6%	2.1%	3.7%	1.7%
Partly work from home	59.8%	52.9%	60.4%	77.2%
**Sector**				
Public	15.9%	21.5%	16.3%	0.6%
Private	76.8%	72.1%	77.8%	87.2%
Self-governing	7.3%	6.5%	5.9%	12.2%
**Industry**				
Buildings and facilities	5.8%	4.4%	7.3%	6.7%
Properties and rental	2.8%	1.7%	3.9%	3.3%
Business services	3.5%	2.5%	5.9%	1.7%
Finance and insurance	5.3%	6.5%	4.5%	3.9%
Trade and transport	18.4%	17.3%	18.3%	21.7%
Industry, raw materials, and supply	21.5%	23.1%	20.8%	18.3%
Information and communication	11.6%	12.1%	9.6%	14.4%
Culture, leisure, and other services	9.8%	9%	7.9%	16.1%
Agriculture, forest, and fishery	1.4%	1.3%	0.6%	3.3%
Public administration, teaching, and health	16.9%	19.6%	18.3%	7.2%
Does not want to say	3.0%	2.7%	3.1%	3.3%
**Number of employees**				
1–49	21.4%	16.5%	12.4%	52.2%
50–99	14.9%	13.1%	17.4%	14.4%
100–249	12.9%	13.8%	13.8%	8.9%
250–499	10.3%	8.3%	11.8%	12.8%
500 or more	40.1%	47.7%	44.1%	11.7%
Does not know/does not want to say	0.5%	0.6%	0.6%	0%

### Controls and Measures

Demographic characteristics are often used as control variables to rule out alternative explanations for the relationship between two constructs. To further investigate the respondents’ experiences and perception of organizational support, we included the following demographic and structural control variables, which may also affect JSA. Each control variable was measured by a single item: age (in intervals), gender (female and male), and management level (top, middle, or line manager). Gender was included as a control variable as several studies show that gender is related to people’s work-life balance, in particular, women are exposed to role conflicts and conflicts between work and family responsibilities (Adisa et al., 2021; Hong et al., 2021) Management level, i.e., seniority was included as a control variable as studies show that seniority is related to work extensification (Worrall and Cooper, 2007). Consequently, we also included age as a control variable.

Based on the literature on POS and JSA we derived the questions for our survey. As the survey was conducted among Danish managers, all questions and statements were translated to Danish. POS was measured with some of the items from the short version of the Survey of Perceived Organizational Support (Eisenberger et al., 1986). A sample item like “The organization values my contribution to its well-being.” was adjusted to the context of distance management (POS4) and reads “I find that my workplace appreciates/recognizes my efforts as a distance manager.” The item “Help is available from the organization when I have a problem,” was split into three questions to identify where the help came from, i.e., employees, top management, and organization (POS 1–3) when WFH. POS and JSA were each measured with 5 items on a five-point Likert scale ranging from 1 (totally disagree) to 5 (totally agree). One item (JSA4) was reversed coded. Both scales had a Cronbach’s alpha >0.7 (see Table 2).

**TABLE 2:** Scale information.

Construct	Measure	Mean (SD)	Factorial loading	Cronbach’s alpha
Perceived organizational support (POS)	POS1: I experience that I get support from my employees in my role as a distance manager	3.83 (1.008)	0.469	0.785
	POS2: I find that I get the support of my top management in my role as a distance leader	3.43 (1.274)	0.819	
	POS3: I experience that I get support from my workplace (e.g., HR/internal support) in my role as distance manager	3.10 (1.394)	0.786	
	POS4: I find that my workplace appreciates/recognizes my efforts as a distance manager	3.53 (1.267)	0.796	
	POS5: My workplace gives me the opportunity to develop my skills as a distance manager (e.g., courses and continuing education)	2.71 (1.457)	0.731	
Job satisfaction (JSA)	JSA1: I like my current role as distance manager	3.16 (1.164)	0.791	0.800
	JSA2: My tasks as a distance manager motivate me	2.91 (1.123)	0.796	
	JSA3: The fact that in my current management job I practice distance management has a positive impact on my development as a leader	3.13 (1.118)	0.700	
	JSA4: The fact that I have to practice distance management in my current job has a negative impact on my well-being (REVERSED)	2.41 (1.281)	0.670	
	JSA5: I experience exciting challenges in my current tasks as a distance manager	3.35 (1.095)	0.702	

**FIGURE 1:**
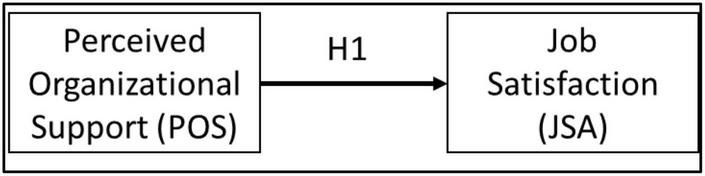
Hypothesized research model.

Based on the literature, we derived the research model as visualized in Figure 1. We investigate whether POS is related to JSA of distance managers. We used PLS-SEM for analyzing the data.

### Data Analyses

We used descriptive analysis to investigate the specific situation of managers WFH. The relationship between POS and JSA (each measured with five items) was calculated using PLS-SEM. We used the recommendations from Hair et al. (2019) to report our findings. In order to investigate possible differences, we controlled for age, gender, WFH status, and manager level. We applied SPSS version 28 and SMART PLS 3.3.9 (Ringle et al., 2015) to analyze the data.

## Results

### Experiences of Becoming a Distance Manager: Work Extensification and Attitude

The survey has allowed us to examine how becoming a distance manager has impacted the managerial work and the experiences of work. When managers worked from home, 57.3% reported that they worked more hours, and 64.8% took fewer breaks. A total of 39.5% of managers had issues with their work-life balance. Nevertheless, 52.7% reported being more productive from home. More specifically, the study has allowed us to assess how their work had extensified. A total of 71.6% of the managers found their work as distance managers more demanding and 64.8% worked more hours. The ANOVA in Table 3 reveals no significant differences between the different management levels regarding the work extensification.

**TABLE 3:** Work extensification of managers at different levels (ANOVA).

Item	Line managers (*N* = 480) Mean (SD)	Middle managers (*N* = 356) Mean (SD)	Top managers (*N* = 180) Mean (SD)	*F*-value	Sign.
It is more demanding to be distance manager than to be manager in the office	3.83 (1.116)	3.87 (1.147)	3.82 (1.192)	0.197	0.821 (n.s.)
I work more hours per day when I work from home	3.59 (1.256)	3.66 (1.265)	3.47 (1.235)	1.420	0.242 (n.s.)
I have problems to separate work and family life when I work from home	2.81 (1.410)	2.94 (1.436)	2.78 (1.327)	1.136	0.322 (n.s.)

During COVID-19, managers also had to manage employees over distance. A total of 67.3% of the surveyed managers would prefer to be a manager from the office than a distance manager. However, 65.9% of all survey participants did not mind having distance management tasks in the future. By applying an ANOVA, we could not find significant differences between the three levels of managers regarding the preference for management from the office or home.

### Experiences of Perceived Organizational Support and the Relation to Job Satisfaction

Regarding POS, 70.6% of managers reported good support from their employees, 50.9% from the top management, and 44.8% got good support from their workplace (HR and internal support). Table 4 shows that the support from the workplace was perceived as above average for POS1–POS4. The agreement was lower for the development of distance management skills in the form of training. Differences between the three different management levels were measured with an ANOVA. Significant differences could only be seen for POS2 and POS5. However, the Eta-squared effect size was only 0.007 in both cases.

**TABLE 4:** ANOVA results for perceived organizational support.

Item	Line managers (*N* = 480) Mean (SD)	Middle managers (*N* = 356) Mean (SD)	Top managers (*N* = 180) Mean (SD)	*F*-value	Sign.
POS1: I experience that I get support from my employees in my role as a distance manager	3.82 (0.993)	3.81 (1.012)	3.88 (1.045)	0.308	0.735 (n.s.)
POS2: I find that I get the support of my top management in my role as a distance leader	3.35 (1.267)	3.48 (1.277)	3.68 (1.270)	3.210	0.041 (sig.)
POS3: I experience that I get support from my workplace (e.g., HR/internal support) in my role as distance manager	3.03 (0.339)	3.21 (1.385)	3.09 (1.542)	1.726	0.179 (n.s.)
POS4: I find that my workplace appreciates/recognizes my efforts as a distance manager	3.43 (1.236)	3.59 (1.247)	3.64 (1.373)	2.572	0.077 (n.s.)
POS5: My workplace gives me the opportunity to develop my skills as a distance manager (e.g., courses and continuing education)	2.59 (1.414)	2.78 (1.471)	2.89 (1.519)	3.555	0.029 (sig.)

**FIGURE 2:**
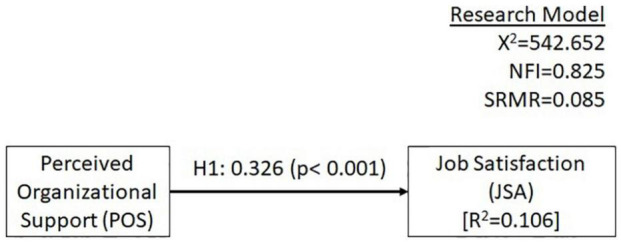
Results of structural model analysis (sample *N* = 1,016).

We conducted a PLS-SEM analysis to check the hypotheses derived from our literature review. Results from our analysis of our structural model on the antecedents of JSA, including path coefficients and their statistical significance, are illustrated in Figure 2. Standard errors were computed by a bootstrapping procedure with 500 re-samples.

The evaluation of the measurement model (outer model) showed that only two loading coefficients had values between 0.6 and 0.7. All others had values above 0.7. The internal consistency of the scales was tested with Cronbach’s alpha, and the values for both constructs were above 0.7 (Table 2). Furthermore, the average variance extraction (AVE) was above 0.5 as recommended [AVE(JSA) = 0.557, AVE(POS) = 0.531]. The heterotrait-monotrait ratio (HTMT) values were below 0.85.

The evaluation of the structural model (inner model) showed that all VIF values were below 3, so no colinearity issues could be found. The Standardized Root Mean Square Residual (SRMR) index of our model is below the recommended upper threshold of 0.1. The recommended criteria of 0.8 for Normed Fit Index (NFI) has also been surpassed by our model. *R*^2^ with a value of 0.106 shows a weak predictive power. The effect size, measured with *f*^2^ was 0.118 showing a weak effect. *Q*^2^ with 0.062 was above 0.

Our hypothesized relationship of a positive relation of POS with JSA of distance managers is substantiated by empirical evidence (β = 0.326, *p* < 0.001).

The following control variables were included in this research: (1) gender, (2) age, and (3) management level. None of these control variables had a significant influence.

## Discussion

### Managerial Work Became Extensified but Managers Also Appreciate Working From Home

The study’s first aim was to explore managers at all organizational levels and their experience in becoming a distance manager. The analysis reveals that acting as a distance manager, independent of managerial level, implied their work became extensified. One of the essential consequences of the managing from home was longer work days and fewer breaks. This is in line with other studies that have found work days to become longer when WFH (Kirchner et al., 2021) where people in the first year of the pandemic worked on average 48 min more per day than before the pandemic (Kost, 2020).

We also found that most managers (67%) prefer to manage from the office, which might be the consequence of work extensification. From the results of this study, the specific reasons managers do not mind managing from home are not evident. However, another study shows that managers appreciate the increased efficiency and control of the work day (Ipsen et al., 2021).

### Managers’ Job Satisfaction Is Positively Related to Perceived Organizational Support

As managers are also employees, we would anticipate that there is a positive correlation between POS and JSA. Similar to employee studies, where it is widely recognized that JSA is linked to organizational support, the same holds true for managers. Since managers’ well-being and JSA impact employee well-being, there is reason to focus on managers as a means to prevent potential problems. This entails that organizations must be aware of the importance and potential of organizational support and consider whether it can be delivered by in-house actors like HRM. This is because the given support or, conversely, the lack of it, has profound implications for the well-being of managers and their JSA.

In the analysis, we included the following control variables: gender, age and management level. However, none of these control variables had a significant influence on the relationship, i.e., there are no differences in the relation of POS on JSA for male/female managers, or managers of different age classes. Moreover, the management level has no influence, differently from what we expected, although managers at different levels have different tasks and responsibilities. Regarding the latter, this is surprising as previous studies show that management level has been related to well-being and stress. Studies show that top managers were least likely to experience stress, although they also tend to work more hours, in contrast to middle or line managers who work fewer hours but are more likely to report higher levels of stress (Worrall et al., 2016). Consequently, the higher the organizational level, the higher the job and life satisfaction whereas the lower organizational levels imply higher job stress and levels of exhaustion (Burke et al., 2009). Though senior managers are met with higher demands in their role and have power, authority, responsibility, and accountability, they report less stress because they also have more control than mangers at lower levels.

### The Support Did Not Come From the Support Functions

We found that managers find it difficult to act as distance managers. This may be due to the extensification of work (Table 3) or the lack of support. In this study, we extended the concept of POS as originated from Eisenberger by looking not only at managerial support but also at support from different levels (HRM, employees, and other managers) (Eisenberger et al., 1986; Rhoades and Eisenberger, 2002). The direction of support in our study is both vertical (from subordinates and higher-level managers) and horizontal (from direct colleagues). To our surprise, by focusing on the direction of POS, our study shows that managers, independent of management level, gender, and age experience POS in the same way. The analysis shows that managers received most support from their own employees whereas the administrative support has been lacking compared to the support from manager peers and employees.

Looking at the concept of POS in previous work, the emphasis is usually on employees, but we widen the view by investigating manager perspectives (Eisenberger et al., 2002; Caesens and Stinglhamber, 2014).

## Conclusion

The COVID-19 pandemic has radically changed the world of work and how it is being managed by creating a distance between the physical workplace and the managers and employees. This quantitative study investigated managers’ well-being and JSA after the first year of working during the pandemic restrictions and, in particular, zooming in on their POS. Analyzing managers’ perception of organizational support in spring 2021, after 1 year in the COVID-19 pandemic, the paper demonstrates that 72% of managers found distance management more demanding and not motivating overall. The hypothesis that POS has a positive effect on managers’ JSA, similarly to employees, proved to be right. However, we could not find any differences for managers on different management levels, as all of them seemed to be challenged and felt supported in a similar way.

The analysis also shows that many managers may experience a dilemma, where the majority (67%) of them would prefer to manage from the office, but at the same time they can continue as distance managers after the pandemic, if needed. The findings also highlight that perceived managerial support has, for managers as for all employees, a positive relation with their JSA.

We argue that to harvest the benefits of telework and WFH post-pandemic, attention must also be paid to the organizational support, how it is conduced and by which actor(s). Consequently, improving support from top management and in-house support functions like HRM can help improve the motivators and JSA for managers. Therefore, it is essential to consider which actions HRM and top managers can take in the workplace going forward with the anticipated transition to telework and WFH. The study adds to the literature on workplaces’ transition to distance management and telework and contributes to understanding the role of POS and managers’ JSA in the transition to become a distance manager.

### Limitations and Avenues for Research

While the strength of this study is the unique focus on managers at different levels, their well-being and JSA during the COVID-19 pandemic, acting as distance managers and their perception of organizational support, collecting data amidst a pandemic implies obvious limitations.

First, as sources of JSA can change over time, the insight from this study is only temporal. It would be essential to examine the support at another point in time and explore whether the managers have the same perception of support or whether their experience results from the point in time. Second, the study was conducted shortly after the second wave of COVID-19, where restrictions were partly lifted (spring 2021), but the pandemic was still ongoing. Therefore, we propose that more national and international studies should be conducted with the same focus post the COVID-19 pandemic, as this would provide an opportunity to validate the results of this study. Specifically, it would be important to replicate the study after the pandemic when new hybrid work settings are implemented across countries with a particular focus on supporting managers during the transition. Following this, studying POS and JSA in a new work setting (WFH) and from a management perspective, we chose to formulate the survey questions ourselves, based on existing questions. Ideally, we could have used existing scales but as these do not take the work situation, WFH, into consideration, we decided to adapt the questions asked from the literature on POS and JSA. Consequently, further studies are suggested to apply the same survey to validate the scales.

Furthermore, we excluded statements about the self-efficacy of managers. Self-efficacy might already lead to high JSA among managers. Organizational support was furthermore not measured objectively (e.g., by number of contacts), but by subjective perceptions of the managers. Therefore, the support could be perceived as low or high independently of the real amount of support. Consequently, to provide an objective insights into the actual practice, future studies should focus on the provided support by different actors, as well as the type, frequency and quality, and on whether the provided support met the managers’ needs. Finally, more demographic factors would provide more insights into the experiences of distance management in distinct groups or situations like seniority or public/private workplaces. Future studies could thus contribute to a better understanding of the effects of hybrid work on managers’ experiences and the role of organizational actors.

Studies have shown that managers’ well-being can have an impact on employee well-being (Skakon et al., 2010; Inceoglu et al., 2018). With the increased use of telework and more people are to WFH, it is thus important for organizations to know how to support managers in their new role as it has the potential to affect their employees. Developing organizational support for managers in a telework context, we suggest that new research on managerial work must entail which actors are central, and what kind of support managers need and how this support should be implemented, including what it entails in relation to the organization. Other studies have pointed to a risk that when managerial capabilities are weak, then organizations do not reach their full potential (Bajorek, 2020). Consequently, if workplaces increase the use of WFH post COVID-19, distance managers must acquire new skills and accept the change in the nature of their job. Accordingly, there is a need for new knowledge about the challenges distance managers face and the development of guidelines on how to support distance managers.

### Practical Implications

In the light of these findings, if workplaces aim to become increasingly flexible, it is essential to take into account the sufficient POS in order to maintain managers JSA. As the distance managers perceived the lowest support from internal support functions, this should be improved in the future in order to enhance well-being. The changes in management locality should align to their skills and preferences (present or acquired). Not taking into account, such skills and preferences may risk increased stress and anxiety and potentially ineffective management and work performance. Consequently, organizations would need to reconsider the support provided to their managers in the transition so that they, the managers, can develop in tandem with the changes and ensure sustainable management.

Our study has shown that distance management is about more than day-to-day management. Organization for Economic Co-operation and Development (OECD) points to the need for businesses to adapt their working practices to address the challenges associated with teleworking and WFH, including ensuring that managers have the skills and tools to coordinate tasks effectively and communicate (OECD, 2021). Our study shows that workplaces should consider how to support their managers in developing these skills and tools. However, telework and WFH are not just a geographical displacement of work affecting people’s well-being and JSA and performance. The future of work should be regarded as an organizational change that includes larger changes than an adaption of work practices and management skills. Introducing new ways of working implies a different view on structures, systems, technologies, and competences. That requires that managers can co-develop with the new organizational design to achieve the organizational goals (Ipsen et al., 2018).

Going forward, HRM will have to play a key role in understanding the extent of corporate change and managers’ challenges and in ensuring that managers receive the necessary support in that change. A recommendation must therefore be made that HRM takes the managers’ abilities and preferences into account and give them the necessary organizational support so that they can develop in tandem with the changes and thrive in the process and thus ensure employee well-being, efficient management of the management task and realization of the company’s goals.

## Data Availability Statement

The data are available from CI, chip@dtu.dk, with the permission of Ledernes Hovedorganisation (DAME). The data that support the findings of this study are available from the corresponding author, CI, upon reasonable request.

## Ethics Statement

Ethical review and approval was not required for the study on human participants in accordance with the local legislation and institutional requirements. The patients/participants provided their written informed consent to participate in this study.

## Author Contributions

CI and KK jointly conceived and designed the study and the questionnaire. KK extracted the data from the surveys and analyzed the data. CI, KK, NA, and MK-M produced the first draft of the manuscript, which included the first draft of the analysis conducted by KK. MK-M audited the interpretation of the data. All authors read, amended drafts of the manuscript, and agreed to the published version of the manuscript.

## Conflict of Interest

The authors declare that the research was conducted in the absence of any commercial or financial relationships that could be construed as a potential conflict of interest.

## Publisher’s Note

All claims expressed in this article are solely those of the authors and do not necessarily represent those of their affiliated organizations, or those of the publisher, the editors and the reviewers. Any product that may be evaluated in this article, or claim that may be made by its manufacturer, is not guaranteed or endorsed by the publisher.
